# Inhibition of cyclooxygenase-2 activity in subchondral bone modifies a subtype of osteoarthritis

**DOI:** 10.1038/s41413-019-0071-x

**Published:** 2019-09-11

**Authors:** Manli Tu, Mi Yang, Nanxi Yu, Gehua Zhen, Mei Wan, Wenlong Liu, Baochao Ji, Hairong Ma, Qiaoyue Guo, Peijian Tong, Li Cao, Xianghang Luo, Xu Cao

**Affiliations:** 1 Department of Orthopaedic Surgery, The Johns Hopkins University School of Medicine, Baltimore, MD 21205 USA; 2 Department of Endocrinology, The First Affiliated Hospital of Nanchang University, 330006 Nanchang, Jiangxi China; 3 Endocrinology Research Center of Xiangya Hospital, Central South University, 410008 Changsha, Hunan China; 4 Department of Orthopedic Surgery, The First Affiliated Hospital of Xinjiang Medical University, 830054 Urumqi, Xinjiang Uygur Autonomous Region China; 5 Department of Orthopedic Surgery, The First Affiliated Hospital of Zhejiang Chinese Medical University, 310006 Hangzhou, Zhejiang Province China

**Keywords:** Pathogenesis, Bone

## Abstract

Osteoarthritis (OA) causes the destruction of joints. Its pathogenesis is still under investigation, and there is no effective disease-modifying therapy. Here, we report that elevated cyclooxygenase-2 (COX-2) expression in the osteocytes of subchondral bone causes both spontaneous OA and rheumatoid arthritis (RA). The knockout of COX-2 in osteocytes or treatment with a COX-2 inhibitor effectively rescues the structure of subchondral bone and attenuates cartilage degeneration in spontaneous OA (STR/Ort) mice and tumor necrosis factor-α transgenic RA mice. Thus, elevated COX-2 expression in subchondral bone induces both OA-associated and RA-associated joint cartilage degeneration. The inhibition of COX-2 expression can potentially modify joint destruction in patients with arthritis.

## Introduction

Arthritis is a joint disorder that affects one or more joints. Osteoarthritis (OA) and rheumatoid arthritis (RA) are the two most common forms of arthritis. OA is characterized mainly by joint pain and stiffness that especially affects weight-bearing joints, such as the knee and hip.^1^ Muscle atrophy and joint deformities appear during the advanced stages.^2^ RA is an autoimmune disease involving chronic synovial inflammation within the joints. Approximately 0.5%–1% of the population has RA, and small joints are affected more frequently than large joints.^3^ RA is characterized by increased levels of inflammatory cytokines, including tumor necrosis factor-α (TNF-α), interleukin-6, and interleukin-1, in the synovial joints.^4^ Currently, arthritis treatment focuses on controlling the symptoms, especially pain; there is no satisfactory prevention or cure. During the end stages of OA and RA, joint replacement is often necessary.^3,5^ OA is a costly and prevalent joint disease. OA is predicted to affect 67 million people in the United States by 2030,^6^ and in developed countries, its treatment costs are between 1.0% and 2.5% of gross domestic product.^7^

Clinically, many OA patients have joint pain and degeneration with no history of trauma or other known causes. In addition to posttraumatic OA, there are other subtypes of OA with different pathogenesis. However, we have not been able to identify the different pathological causes of the subtypes of OA. Without identifying OA patients based on specific pathogenesis, it is difficult to develop disease-modifying therapies for clinical trials. Studying spontaneous OA in animals offers a unique opportunity to investigate the pathogenesis of nonposttraumatic OA. The STR/Ort mouse strain is an inbred substrain of STR/N mice.^8^ The origin of STR/Ort mice can be traced to 1951.^9^ Notably, the STR/Ort strain is prone to developing OA, which is characterized by subchondral bone sclerosis, osteophyte formation, and articular cartilage degeneration.^8^ It is worth mentioning that male mice have a higher incidence of OA than female mice.^8^ Histologically, the lesions of STR/Ort mice closely resemble those observed in human OA. Previous studies have shown that OA lesions often develop in the medial tibia plateau of the knee joint rather than the lateral plateau.^8^ Thus, STR/Ort mice are a promising model for studying the potential factors involved in spontaneous OA.

Cyclooxygenase-2 (COX-2) inhibitors selectively block the COX-2 enzyme and have been used to treat OA and RA with a low risk of adverse gastrointestinal effects.^10^ Blocking this enzyme impedes the production of prostaglandin (PG) E2, which is often the cause of pain and swelling during inflammation.^11^ PGs are enzymatically derived metabolites of polyunsaturated fatty acids, such as arachidonic acid. The crucial limiting step in PG synthesis is the enzyme that catalyzes the COX and peroxidase reaction. There are two isoforms of COX: COX-1 is expressed at relatively stable levels in most tissues and is considered constitutive, whereas COX-2 is an inducible isoform that is generally expressed at very low levels in most tissues but can be induced to high levels by multiple factors.^11^ COX-2 is more efficient than COX-1 at producing PGs.^12^ As the most widely produced prostanoid in the human body, PGE2 is a well-known regulator of bone formation.^12^ Our previous study demonstrated that PGE2 activates PGE2 receptor 4 (EP4) in sensory nerves to regulate bone formation by inhibiting sympathetic activity throughout the central nervous system.^13^ We have also demonstrated that uncoupled bone formation in subchondral bone induced by active TGF-β initiates the pathological changes of trauma-induced OA.^14^ COX-2 inhibitors have been mainly used in the clinic for their anti-inflammatory and analgesic effects; however, the potential role of COX-2 in the pathogenesis of OA and the role of its inhibitors as disease-modifying treatments have not been studied. In this study, we explored the effect of COX-2 on subchondral bone and articular cartilage during the progression of arthritis. The knockout of COX-2 in osteocytes or treatment with a COX-2 inhibitor attenuated cartilage degeneration in spontaneous OA and TNF-α transgenic RA mice.

## Result

### Aberrant subchondral bone formation in spontaneous OA mice

**Fig. 1:**
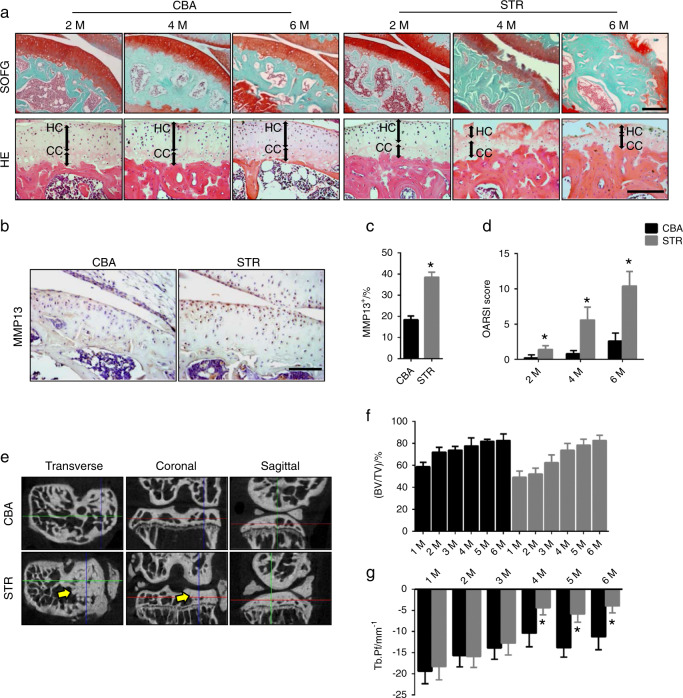
Aberrant subchondral bone formation in spontaneous OA mice. **a** Top: safranin O and fast green staining of sagittal sections of the medial tibial subchondral bone from STR/Ort (STR) and CBA control mice at 2, 4, and 6 months of age. Proteoglycan (red) and bone (blue). Scale bar, 200 μm. Bottom: hematoxylin and eosin staining of the subchondral bone plate and cartilage. The hyaline cartilage (HC) and calcified cartilage (CC) thicknesses are indicated by double-headed arrows. Scale bars, 100 μm. **b** Immunohistochemical staining for MMP13 (brown) in the articular cartilage of STR/ORT and CBA mice at 4 months of age. Scale bars, 100 μm. **c** Quantitative analysis of the proportion of MMP13^+^ cells in the articular cartilage of STR/ORT and CBA mice at 4 months of age. *N* = 5 mice in each group from three independent experiments. **d** Osteoarthritis Research Society International scores of STR/ORT and CBA control mice at 2, 4, and 6 months of age. *N* = 5 mice in each group from three independent experiments. **e** Representative μCT images of transverse, coronal, and sagittal views of the tibial subchondral bone of 4-month-old STR/ORT and CBA mice. **f**, **g** Quantitative analysis of the bone volume (BV)/tissue volume (TV) ratio **f** and trabecular pattern factor (Tb.Pf) **g** in subchondral bone, as determined by μCT analysis. *N* = 6 mice in each group from three independent experiments. All data are shown as the mean ± standard deviation. *P < 0.05 compared with the CBA control group at the corresponding time points. Statistical significance was determined by Student’s *t*-test

To examine the pathogenesis of spontaneous OA, we sectioned the knee joints of STR/Ort mice at different ages for immunostaining analysis. The results showed that, relative to age-matched control CBA mice, significant proteoglycan loss occurred in the deeper zone of cartilage adjacent to subchondral bone from 4 months of age and was aggravated over time (Fig. 1a). The tidemark moved up because of the decreased thickness of the hyaline cartilage zone and the increased calcified cartilage layer, as shown by hematoxylin and eosin staining (Fig. 1a). The percentage of MMP13^+^ chondrocytes increased significantly in 4-month-old STR/Ort mice (Fig. 1b, c), suggesting that the articular cartilage degenerated in STR/Ort mice. Osteoarthritis Research Society International (OARSI) scores^15^ showed that significant articular cartilage degeneration started at 4 months of age in STR/Ort mice and progressed gradually relative to that in the age-matched CBA mice (Fig. 1d).

**Fig. 2:**
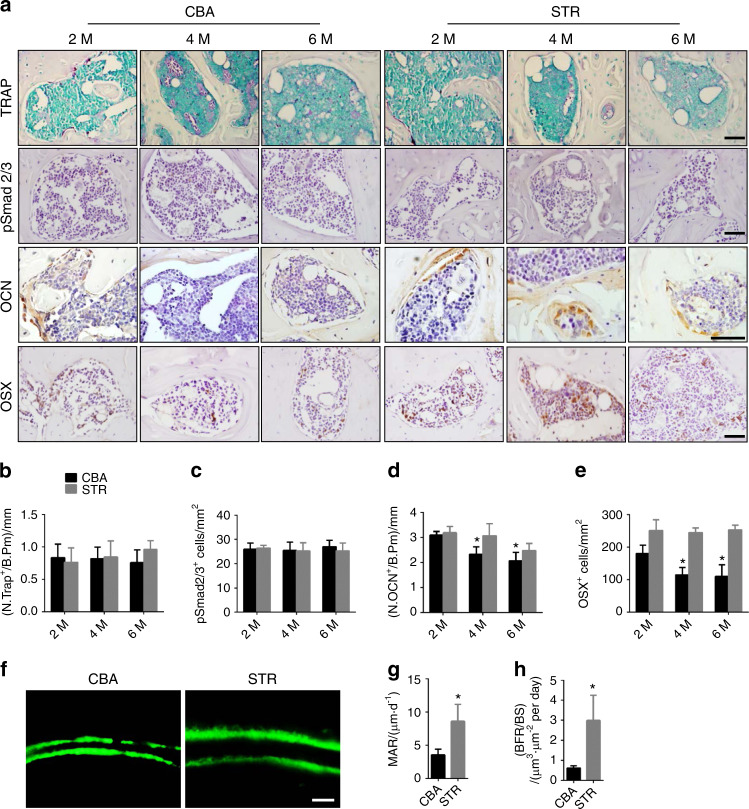
Uncoupled subchondral bone remodeling is not involved in spontaneous osteoarthritis. **a** Images of TRAP staining and immunohistochemical staining of pSmad2/3^+^ cells, osteocalcin^+^ cells, and osterix^+^ cells (brown) in the subchondral bone of STR/ORT mice and CBA controls at 2, 4, and 6 months of age. Scale bars, 50 μm. **b** Quantitative analysis of TRAP^+^ cells on the surface of subchondral bone from STR/ORT mice and CBA controls. *N* = 5 mice in each group from three independent experiments. **c** Quantitative analysis of pSmad2/3^+^ cells in the subchondral bone of STR/ORT mice and CBA controls. *N* = 5 mice in each group from three independent experiments. **d** Quantitative analysis of osteocalcin^+^ cells in the subchondral bone of STR/ORT and CBA mice. *N* = 5 mice in each group from three independent experiments. **e** Quantitative analysis of osterix^+^ cells in the subchondral bone of STR/ORT and CBA mice. *N* = 5 mice in each group from three independent experiments. **f**–**h** Representative images of calcein double labeling of subchondral bone **f** and the quantification of the mineral apposition rate (MAR) **g** and bone formation rate (BFR) per bone surface (BS) **h** in STR/ORT mice and CBA controls at 6 months of age. Scale bar, 25 μm. *N* = 3 per group. All data are shown as the mean ± standard deviation. *P < 0.05, compared with the CBA control group at the corresponding time points. Statistical significance was determined by Student’s *t*-test

We examined tibial subchondral bone using three-dimensional microcomputed tomography (µCT) analysis because uncoupled subchondral bone formation causes OA.^14^ Cross-sectional, coronal, and sagittal views of tibial subchondral bone showed an uneven distribution of bone mass in STR/Ort mice relative to CBA controls, indicating abnormal bone formation (Fig. 1e). The subchondral bone volume (BV)/tissue volume (TV) ratio increased each month by more than 10% in STR/Ort mice, whereas the BV/TV ratio in CBA controls remained relatively steady after puberty (Fig. 1f). Furthermore, the increase in trabecular pattern factor (Tb.Pf) indicated a decrease in subchondral bone connectivity in STR/Ort mice (Fig. 1g). Increases in the number of osteoclasts and transforming growth factor-β (TGF-β) activity in subchondral bone induce traumatic OA in mice that undergo anterior cruciate ligament transection.^14^ However, as in CBA mice, there were no changes in tartrate-resistant acid phosphatase (TRAP)^+^ osteoclasts or pSmad2/3^+^ cells, which represent TGF-β signaling, in STR/Ort mice of different ages (Fig. 2a–c). Interestingly, immunostaining showed that the number of osteocalcin (OCN)^+^ osteoblasts and osterix^+^ osteoprogenitors increased significantly in the subchondral bone marrow from 4 months of age in STR/Ort mice relative to CBA mice (Fig. 2a, d, e), indicating osteoblastic bone formation. The increase in the bone formation rate and mineral apposition rate in calcein double-labeled bone confirmed subchondral bone formation (Fig. 2f–h). Therefore, the pathogenesis of aberrant subchondral bone formation in spontaneous OA is not associated with the elevated number of osteoclasts or transforming growth factor-β activity in traumatic OA.

### Elevated COX-2 activity in subchondral bone in spontaneous OA

**Fig. 3:**
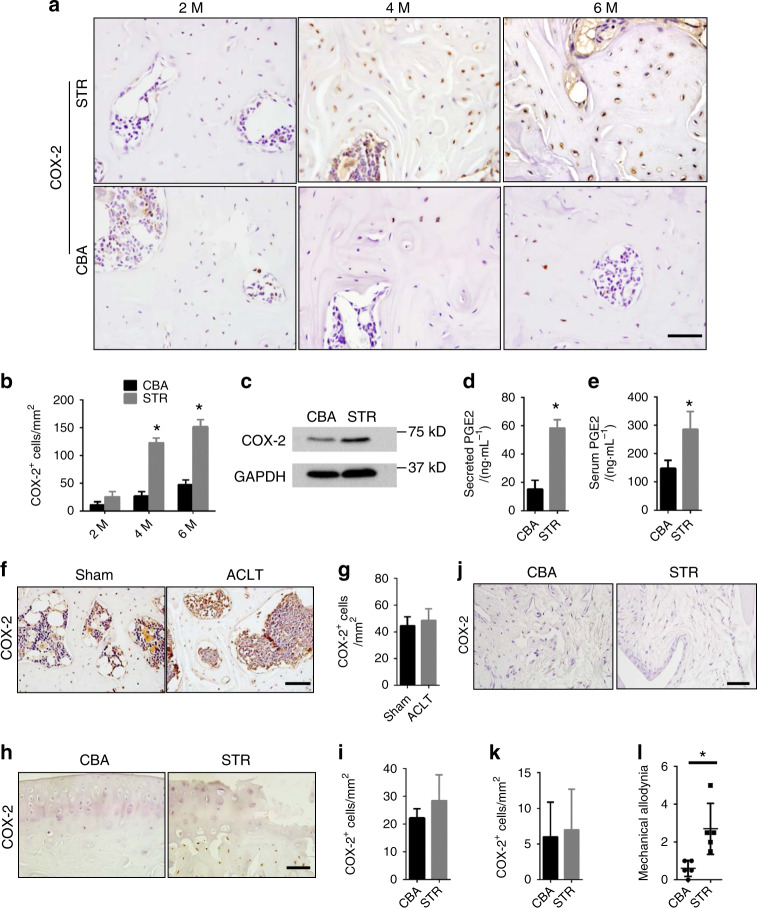
Elevated COX-2 activity in subchondral bone in spontaneous OA. **a** Immunohistochemical staining for COX-2 (brown) in the subchondral bone of STR/ORT and CBA mice at 2, 4, and 6 months of age. Scale bars, 50 μm. **b** Quantitative analysis of COX-2^+^ cells (per mm^2^) in the subchondral bone of STR/ORT and CBA mice. *N* = 5 mice in each group from three independent experiments. **c** Western blot analysis of COX-2 protein expression in osteocytes isolated from the subchondral bone of STR/ORT and CBA mice at 4 months of age. **d** PGE2 levels in the medium used to culture osteocytes isolated from the subchondral bone of 4-month-old STR/ORT and CBA mice. *N* = 5 mice in each group from three independent experiments. **e** Serum PGE2 levels in 4-month-old STR/ORT and CBA mice. *N* = 5 mice in each group from three independent experiments. **f** and **g** Immunohistochemical staining **f** and quantitative analysis **g** of COX-2^+^ cells (brown) in the subchondral bone of C57BL/6 mice 30 days after sham operation or anterior cruciate ligament transection (ACLT) surgery. Scale bar, 50 μm. *N* = 5 mice in each group from three independent experiments. **h** and **i** Immunohistochemical staining **h** and quantitative analysis **i** of COX-2^+^ cells (brown) in the articular cartilage of STR/ORT and CBA mice at 4 months of age. Scale bars, 50 μm. *N* = 5 mice in each group from three independent experiments. **j** and **k** Immunohistochemical staining **j** and quantitative analysis **k** of COX-2^+^ cells (brown) in the synovial membrane of STR/ORT and CBA mice at 4 months of age. Scale bars, 50 μm. *N* = 5 mice in each group from three independent experiments. **l** Quantitative analysis of mechanical allodynia in 4-month-old STR/ORT and CBA mice, as measured by the foot-lift response frequency to stimulation with a 0.008-g von Frey filament. *N* = 5 mice in each group from three independent experiments. All data are shown as the mean ± standard deviation. *P < 0.05. Statistical significance was determined by Student’s *t*-test

Because PGE2 stimulates bone formation without stimulating osteoclast bone remodeling,^12^ we examined whether COX-2 is elevated in subchondral bone formation in STR/Ort mice. Immunostaining showed a significant increase in the number of COX-2^+^ osteocytes in subchondral bone starting at 4 months of age (Fig. 3a, b). COX-2 expression in nonsubchondral bone osteocytes was also increased (Supplementary Fig. 1a–d). COX-2 protein levels in subchondral bone osteocytes were elevated in STR/Ort mice (Fig. 3c), and the amount of PGE2 secreted by osteocytes in STR/Ort mice increased significantly (Fig. 3d). The serum level of PGE2 in 4-month-old STR/Ort mice was twice as high as that in CBA controls (Fig. 3e). There was no increase in COX-2 expression in subchondral bone in traumatic OA mice (Fig. 3f, g) or in articular cartilage chondrocytes or synovial cells in STR/Ort mice compared with controls at 4 months of age (Fig. 3h–k). A high level of COX-2 was associated with joint pain, as demonstrated by a higher response rate exhibited by STR/Ort mice in the von Frey test (Fig. 3l). Collectively, these results indicate that elevated COX-2 expression in subchondral bone is associated with spontaneous OA but not traumatic OA of mechanically unstable joints.

### Elevated COX-2 activity in subchondral bone in genetically modified mice with RA and both human OA and RA

**Fig. 4:**
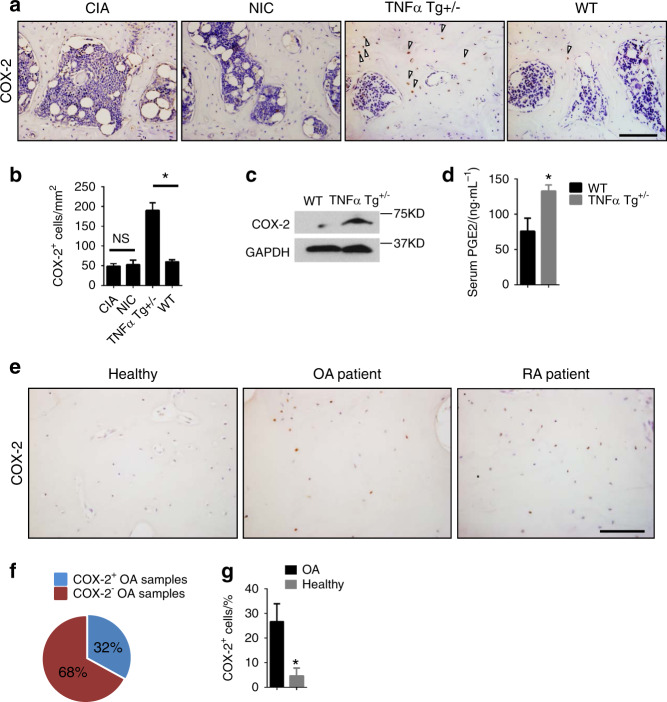
Elevated COX-2 activity in subchondral bone in genetically modified mice with RA and both human OA and RA. **a** and **b** Immunohistochemical staining **a** and quantitative analysis **b** of COX-2^+^ cells (brown) in the subchondral bone of mouse models of type II collagen-induced arthritis (CIA) and their nonimmunized controls (NIC) and in TNF-α transgenic mice (TNF-α Tg^+/−^) and their wild type (WT) controls at 4 months of age. Scale bars, 100 μm. *N* = 5 mice in each group from three independent experiments. **c** Western blot analysis of COX-2 protein expression in osteocytes isolated from the subchondral bone of TNF-α Tg^+/−^ mice and their WT controls at 4 months of age. **d** Serum PGE2 levels in 4-month-old TNF-α Tg^+/−^ mice and their WT controls. **e** Immunohistochemical staining for COX-2 in the subchondral bone of OA patients, RA patients, and their healthy controls. Scale bars, 100 μm. **f** The proportion of subchondral bone samples with high COX-2 expression (more than 20% osteocytes were considered COX-2^+^). **g** The percentage of COX-2^+^ osteocytes in subchondral bone samples with high COX-2 expression from 13 OA patients and 6 healthy controls. All data are shown as the mean ± standard deviation. *P < 0.05. Statistical significance was determined by Student’s *t*-test

We also examined COX-2 levels in subchondral bone in two different mouse models of RA. Interestingly, COX-2 levels were increased in TNF-α transgenic RA mice (TNF-α Tg^+/−^) but not in mice with type II collagen-induced RA (CIA) compared with controls (Fig. 4a, b). Western blot analysis confirmed higher levels of COX-2 expression in subchondral bone in TNF-α transgenic mice (Fig. 4c). As expected, the serum level of PGE2 was also increased (Fig. 4d), as was the number of osterix^+^ osteoprogenitors and TRAP^+^ osteoclasts in subchondral bone in TNF-α transgenic mice (Supplementary Fig. 2a, b, d). There was no difference in the number of OCN^+^ osteoblasts in subchondral bone in TNF-α transgenic mice compared to WT controls (Supplementary Fig. 2a, c). Moreover, we evaluated the expression of COX-2 in the subchondral bone of the knee in human OA and RA patients. Approximately one-third of the 43 human OA knee samples and all of the 9 knee samples from RA patients showed elevated COX-2 expression relative to that shown by healthy controls (Fig. 4e, f). The percentage of COX-2-positive cells was as high as 26% in the knee samples in which COX-2 expression was upregulated (Fig. 4g). Elevated COX-2 levels in spontaneous OA mice, genetically modified mice with RA, and human OA and RA specimens reveals that COX-2 in subchondral bone is a potential factor involved in the pathological processes of both OA and RA.

### Inhibition of COX-2 or the conditional knockout of COX-2 in osteocytes attenuates the progression of RA

**Fig. 5:**
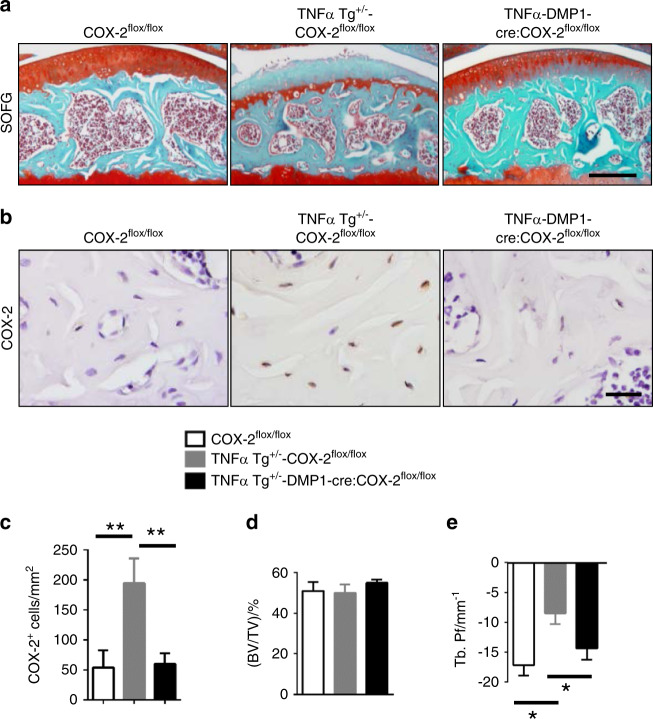
Inhibition of COX-2 attenuates the progression of RA. **a** Safranin O and fast green staining of sagittal sections of the medial tibial subchondral bone of COX-2^flox/flox^, TNF-α Tg^+/−^ -COX-2^flox/flox^, and TNF-α Tg^+/−^ DMP1-Cre:COX-2^flox/flox^ mice. Scale bars, 200 μm. **b** and **c** Immunohistochemical staining **b** and quantitative analysis **c** of COX-2^+^ cells (brown) in the subchondral bone of COX-2^flox/flox^, TNF-α Tg^+/−^ -COX-2^flox/flox^, and TNF-α Tg^+/−^ DMP1-Cre:COX-2^flox/flox^ mice. Scale bars, 25 μm. *N* = 5 mice in each group from three independent experiments. **d** and **e** Quantitative analysis of the bone volume (BV)/tissue volume (TV) ratio **d** and trabecular pattern factor (Tb.Pf) **e** in subchondral bone of COX-2^flox/flox^, TNF-α Tg^+/−^ -COX-2^flox/flox^, and TNF-α Tg^+/−^ DMP1-Cre:COX-2^flox/flox^ mice, as determined by μCT analysis. *N* = 5 mice in each group from three independent experiments. All data are shown as the mean ± standard deviation. *P < 0.05; **P < 0.01. Statistical significance was determined by ANOVA

Dentin matrix protein 1 (DMP1) is a matrix protein that is expressed in odontoblasts, preosteocytes, and osteocytes. DMP1-Cre transgenes are widely used to target osteocytes.^16,17^ To specifically knock out COX-2 in osteocytes in TNF-α transgenic mice, we crossed DMP1-Cre mice with TNF-α transgenic mice, and their progeny DMP1-Cre:TNF-α transgenic mice were further crossed with COX-2^flox/flox^ mice. Articular cartilage degeneration was attenuated in COX-2^−**/−**^ RA (TNF-α DMP1-Cre: COX-2^−/−^) mice relative to TNF-α COX-2^flox/flox^ mice (Fig. 5a). As expected, the number of COX-2^+^ osteocytes decreased significantly (Fig. 5b, c), and the number of osteocalcin^+^ osteoblasts and TRAP^+^ osteoclasts did not change significantly compared with that in controls (Supplementary Fig. 3a–c), but the number of osterix^+^ osteoprogenitors in COX-2^−**/−**^ RA mice decreased relative to that in controls (Supplementary Fig. 3a, d). The quality of subchondral bone improved significantly, as evidenced by increased connectivity with no change in BV (Fig. 5d, e).

To investigate whether COX-2 inhibitors have a disease-modifying effect by rescuing subchondral bone structure in genetically modified mice with RA, we gavage-fed the COX-2 inhibitor celecoxib to TNF-α transgenic mice at a dose of 8 mg·kg^–1^ daily for 4 weeks. The serum level of PGE2 decreased significantly upon the administration of the COX-2 inhibitor (Supplementary Fig. 4b). Importantly, the degeneration of articular cartilage was ameliorated (Supplementary Fig. 4a), and the number of osterix^+^ osteoprogenitors in the subchondral bone marrow decreased upon COX-2 inhibitor administration (Supplementary Fig. 4e, h). However, the number of osteocalcin^+^ osteoblasts and TRAP^+^ osteoclasts did not change significantly in inhibitor-treated mice (Supplementary Fig. 4e–g). The quality of subchondral bone improved significantly, as evidenced by increased connectivity with no change in BV (Supplementary Fig. 4c, d). Thus, the fact that the inhibition or knockout of COX-2 attenuated RA confirms the role of COX-2 in subchondral bone during the progression of RA.

### Inhibition of COX-2 attenuates spontaneous OA

**Fig. 6:**
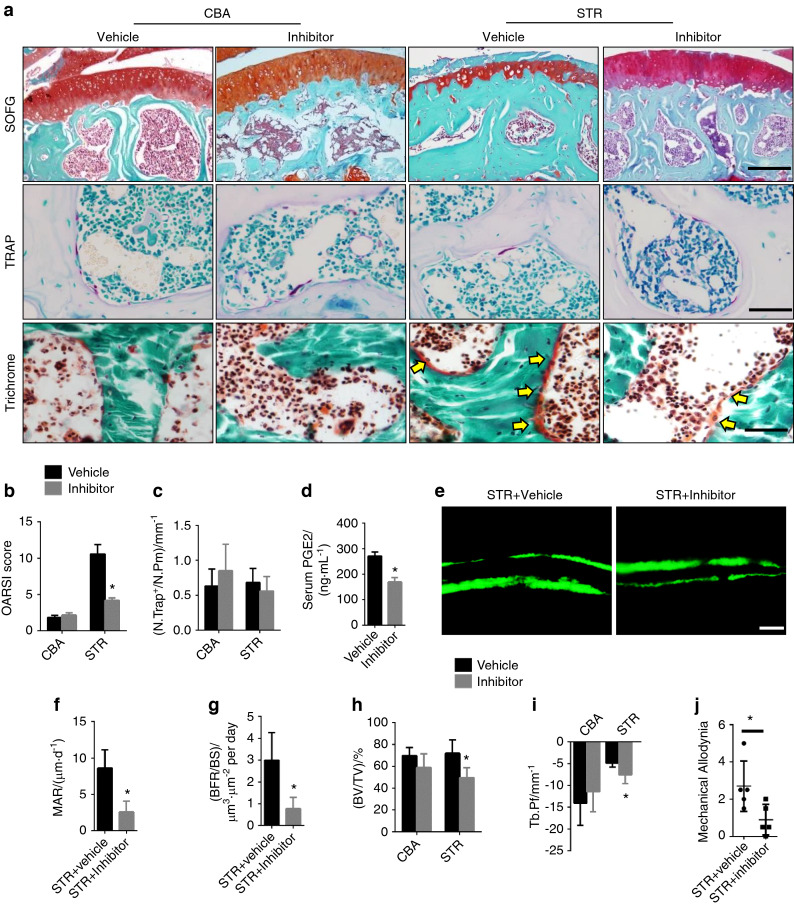
Inhibition of COX-2 attenuates spontaneous OA progression in STR/ORT mice. **a** Top: safranin O and fast green staining of the subchondral bone of STR/ORT mice and CBA control mice treated with vehicle or a COX-2 inhibitor. Proteoglycan (red) and bone (blue). Scale bar, 200 μm. Middle: representative images of TRAP staining in the subchondral bone of STR/ORT and CBA mice treated with vehicle or a COX-2 inhibitor. Scale bars, 50 μm. Bottom: trichrome staining of the subchondral bone of STR/ORT and CBA mice treated with vehicle or a COX-2 inhibitor. Scale bars, 50 μm. **b** OARSI scores of STR/ORT mice and CBA control mice treated with vehicle or a COX-2 inhibitor. *N* = 5 mice in each group from three independent experiments. **c** Quantitative analysis of TRAP^+^ cells on the bone surface in STR/ORT mice and CBA control mice treated with vehicle or a COX-2 inhibitor. *N* = 5 mice in each group from three independent experiments. **d** Serum PGE2 levels in STR/ORT mice and CBA control mice treated with vehicle or a COX-2 inhibitor. *N* = 5 mice in each group from three independent experiments. **e**–**g** Representative images of calcein double labeling of subchondral bone **e** and the quantification of the mineral apposition rate (MAR) **f** and bone formation rate (BFR) per bone surface (BS) **g** in STR/ORT mice treated with vehicle or a COX-2 inhibitor. Scale bar, 25 μm. **h** and **i** Quantitative analysis of the bone volume (BV)/tissue volume (TV) ratio **h** and trabecular pattern factor (Tb.Pf) **i** in subchondral bone, as determined by μCT analysis. *N* = 5 mice in each group from three independent experiments. **j** Quantitative analysis of mechanical allodynia in STR/ORT mice treated with vehicle or a COX-2 inhibitor, as measured by the foot-lift response frequency to stimulation with a 0.008-g von Frey filament. *N* = 5 mice in each group from three independent experiments. All data are shown as the mean ± standard deviation. *P < 0.05. Statistical significance was determined by Student’s *t*-test

Finally, we examined whether the inhibition of COX-2 also attenuates spontaneous OA. Three-month-old STR/Ort mice and CBA controls were gavage-fed the COX-2 inhibitor celecoxib at different doses once a day for 4 weeks. Proteoglycan loss and the calcification of articular cartilage were effectively attenuated by 8 mg·kg^–1^ COX-2 inhibitor in STR/Ort mice relative to controls, as indicated by safranin O and fast green staining (Fig. 6a); the OARSI score for articular cartilage also improved significantly (Fig. 6b). The serum level of PGE2 decreased significantly in STR/Ort mice treated with the COX-2 inhibitor, but the number of TRAP^+^ osteoclasts did not change (Fig. 6a, c, d). Subchondral bone osteoid formation decreased significantly on the bone surface upon treatment with the COX-2 inhibitor, as indicated by trichrome staining (Fig. 6a). Calcein double-labeling confirmed that the subchondral bone formation rate and mineral apposition rate were decreased in STR/Ort mice treated with the COX-2 inhibitor (Fig. 6e–g). The trabeculae connectivity and microarchitecture of subchondral bone improved significantly (Fig. 6h, i). Interestingly, von Frey testing showed that pain sensitivity decreased after the administration of the COX-2 inhibitor in spontaneous OA mice (Fig. 6j), indicating that a decrease in PGE2 reduces pain. In parallel, CBA mice with the same genetic background as the STR/Ort mice were treated with the COX-2 inhibitor and exhibited no significant changes in subchondral bone or articular cartilage (Fig. 6a–d). Taken together, these results suggest that the elevation of COX-2 levels in subchondral bone causes spontaneous OA.

## Discussion

OA is a highly heterogeneous disease with important contributing factors, including heredity, prior injury, and abnormal joint alignment. In this study, we found that elevated levels of COX-2 stimulated aberrant subchondral bone formation in the development of OA. This newly identified pathogenic process of OA may potentially represent a subtype of OA. Indeed, 1/3 of OA patients, including RA patients, showed high levels of COX-2 expression. Identifying the potential factors that are associated with OA has been one of the major efforts in elucidating OA.^18–21^ Knee joint destruction in various animals models of RA may be caused by either an excessive amount of active TGFβ or elevated COX-2 expression in subchondral bone.^4,22^ It appears that different factors may be associated with a single OA pathogenesis and vice versa. Therefore, understanding the pathogenic mechanism to define the subtypes of OA would be helpful for developing disease-modifying treatments.

The STR/ORT strain was first isolated by Strong by crossing the CBA strain, the N strain, the J strain as a spontaneous OA model, and the K strain to experimentally imitate the genetic variability of humans in mice.^23^ STR/Ort mice were derived from the parent STR/1N strain, which was isolated by Dr. George E. Jay, Jr. in 1951 as a spontaneous piebald mutant of STR/ORT that arose in the F29 generation.^9^ The STR/Ort line is a well-recognized model of a natural form of OA. These mice spontaneously develop OA with high incidence and severity, and they exhibit human-like characteristics, such as proteoglycan loss, articular cartilage fibrillation, active extracellular matrix degradation, osteophyte formation, and subchondral sclerosis.^8^ It has been reported that STR/Ort mice have a novel high bone mass phenotype associated with elevated osteoblast activity.^24^ In our study, we confirmed that the disruption of subchondral bone structure is consistent with the high bone mass phenotype of STR/Ort mice (Fig. 1e–g, Supplementary Fig. 5a–f). Six hundred twenty-one genes are associated with both osteophyte formation and cartilage damage in STR/Ort joints; however, no specific gene loci have been found to be responsible for the onset of OA.^25^ A higher level of COX-2 enzymatic activity is unlikely to be derived from a COX-2 mutation, whereas mutations in the COX promoter may lead to the high levels of its expression that are responsible for architecture disruption in subchondral bone in STR/Ort mice. In recent years, genome-wide association studies have identified a number of common sequence variants that confer small to moderate effects.^19,20,26^ A common promoter variant (−765G → C) of the COX-2/PG-endoperoxide synthase 2 (*PTGS2*) gene that represses COX-2 expression is associated with a lower risk of hip and knee OA.^27–29^ A genome-wide association study conducted by Valdes and colleagues identified rs4140564, which maps 5′ to the COX-2/*PTGS2* gene, as the only single nucleotide polymorphism significantly associated with a risk of knee OA in all cohorts from the United Kingdom, the United States, and the Netherlands.^21^ In our research, we found that increased COX-2 expression in the osteocytes of subchondral bone induces both spontaneous OA and transgenic RA in mice. There was a high level of COX-2 expression in one-third of the 43 OA patients and in all 9 late-stage RA patients. Our results indicate that the elevated expression of COX-2 in subchondral bone may represent a subtype of OA.

Subchondral bone, which has the ability to eliminate loading from cartilage, plays an important role in joint degradation in OA.^30,31^ Our previous research demonstrated that a high level of active TGF-β can recruit progenitor cells and result in uncoupled bone formation in subchondral bone and the initiation of the pathological changes of OA.^14^ We also demonstrated that this pathological process in subchondral bone is involved in a model of type II CIA.^22^ COX-2/PGs are known multifunctional regulators of bone metabolism, and PGE2 can stimulate bone formation.^12,32^ Recently, we found that (PGE2) secreted by osteoblastic cells activates PGE2 receptor 4 (EP4) in sensory nerves to regulate bone formation by inhibiting sympathetic activity throughout the central nervous system.^13^ In the current study, we focused on the role of COX-2 in the metabolism and structure of subchondral bone during the onset of spontaneous OA, which is independent of inflammatory and immunomodulatory pathways in diseased joints.

COX-2 inhibitors have been used to relief pain associated with arthritis. However, the disease-modifying function of COX-2 inhibitors is not clear. Clinically, COX inhibitors (as nonsteroidal anti-inflammatory drugs), along with glucocorticoids, are used as analgesics and antipyretics for the treatment of RA and OA, and at higher doses, for anti-inflammation.^11^ However, the disease-modifying effect of COX inhibitors on arthritis is still unknown. The gastrointestinal side effects of nonselective COX inhibitors and the cardiovascular side effects of selective COX-2 inhibitors limit their application, and the lowest dose and shortest term are recommended for clinical use.^33^ We demonstrated that high levels of COX-2 expression in osteocytes induce abnormal bone formation in subchondral bone, therefore accelerating cartilage degeneration in the onset of spontaneous OA and RA. A reduction in COX-2 results from treatment with a COX-2 inhibitor at a dose of one-eighth to one-quarter of the currently used clinical dose for 4 weeks reversed subchondral bone structural damage, and attenuated cartilage degeneration in spontaneous OA and RA mouse models. Therefore, for OA or RA patients with high levels of COX-2 expression, a low dose of a COX-2 inhibitor may modify the disease and relieve pain.

## Materials and methods

### Human subjects

Human subchondral bone samples were obtained from 43 patients with OA and 9 patients with RA that underwent knee joint replacement or open reduction and internal fixation at The First Affiliated Hospital of Xinjiang Medical University or The Johns Hopkins University School of Medicine. All subjects were screened based on responses to a detailed questionnaire, disease history, and physical examination.

### Mice

Mouse studies were conducted in the animal facility of The Johns Hopkins University School of Medicine, and the procedures were performed under a protocol approved by the Institutional Animal Care and Use Committee of The Johns Hopkins University (Baltimore, MD, USA). STR/Ort mice were purchased from Harlan Laboratories (Frederick, MD, USA), CBA/J mice and DMP1-Cre mice were purchased from the Jackson Laboratory (Bar Harbor, ME, USA), TNF-α transgenic (hemizygous) mice were obtained from Taconic Biosciences (Hudson, NY, USA), and COX-2^flox/flox^ mice were provided by Harvey Herschman, Ph.D., at the University of California-Los Angeles (Los Angeles, CA, USA).

To knock out COX-2 in the osteocytes of TNF-α transgenic mice, we first crossed TNF-α transgenic mice with DMP1-Cre mice to create DMP-1Cre:TNF-α transgenic offspring. Then, DMP-1Cre:TNF-α transgenic mice were crossed with COX-2^flox/flox^ mice to create TNF-α DMP1-Cre:COX-2^flox/flox^ experimental mice and COX-2^flox/flox^ littermate controls.

CIA procedures were performed on 2-month-old mice using the methods described by Brand and colleagues.^34^ For the time-course experiments, CIA mice or nonimmunized controls were euthanized 100 days after initial immunization.

### Cell culture

We isolated primary osteocytes from different mouse models using a protocol described previously.^35^ We collected subchondral bone from mice by carefully removing the attached soft tissue and then washing the bones with α-minimum essential medium (MEM) + 10% penicillin and streptomycin to remove contaminants. We cut the bone into 1–2-mm sections and incubated the bone pieces in warmed collagenase solution (4 mg·mL^–1^ type IA collagenase in α-MEM) three times at 25 °C for 25 min. Then, we incubated the bone pieces in warmed collagenase solution (4 mg·mL^–1^ type IA collagenase in α-MEM) and ethylenediaminetetraacetic acid (EDTA) solution (5 mmol·L^–1^ EDTA solution in Dulbecco’s phosphate-buffered saline [PBS] with 1% bovine serum albumin) two times at 25 °C. Alternatively, we aspirated the solution and retained it for cell plating. The isolated primary osteocytes and minced bone pieces were cultured in primary bone cell culture medium (α-MEM with 5% fetal bovine serum, 5% calf serum, and 1% penicillin and streptomycin) in collagen-coated plates. We collected protein and the culture medium for Western blot and enzyme-linked immunosorbent assay (ELISA) analysis at 72 h.

### ELISA and western blot

We detected the levels of serum and medium PGE2 using a PGE2 ELISA kit (514010, Cayman Chemical, Ann Arbor, MI, USA) according to the manufacturer’s instructions. Total cell lysates were separated by sodium dodecyl sulfate polyacrylamide gel electrophoresis (SDS–PAGE) and blotted on polyvinylidene fluoride membranes (Millipore, Sigma, Temecula, CA, USA). The membranes were blocked with 5% milk (170-6404, Bio-Rad Laboratories, Inc., Hercules, CA, USA), incubated with a specific antibody against COX-2 (ab15191, 1:20 000, Abcam, Cambridge, MA, USA), and reprobed with an appropriate horseradish peroxidase-conjugated secondary antibody. The blots were developed using a SuperSignal West Femto Maximum Sensitivity Substrate Kit (QJ222305, Thermo Fisher Scientific, Inc., Waltham, MD, USA) and exposed to X-ray film.

### µCT analysis

Knee joints were dissected from mice, the attached muscle was carefully removed, and the tissues were fixed for 4 h with 4% paraformaldehyde at 4 °C, and washed three times with ice-cold PBS. We then performed µCT analysis using high-resolution μCT (Skyscan 1172, Bruker microCT, Kontich, Belgium). The scanner was set at a voltage of 65 kV, a current of 154 μA, and a resolution of 5.8 μm per pixel. Image reconstruction software (NRecon, version 1.6, Bioz, Inc., Palo Alto, CA, USA), data analysis software (CT Analyser, version 1.9, Bruker microCT) and three-dimensional model visualization software (μCT Volume, version 2.0, Bruker microCT) were used to analyze the parameters of the distal femoral metaphyseal trabecular bone. We selected the subchondral trabecular bone as the region of interest for analysis. The trabecular BV per TV and Tb.Pf were measured.

### Histochemistry and histomorphometry

Knee joints were dissected from mice, the attached muscle was carefully removed and the tissues were fixed overnight with 10% formalin at 4 °C. After washing three times with ice-cold PBS, the samples were decalcified at 4 °C using 10% EDTA (pH 7.4) for 21 days and then embedded in paraffin. Four-micrometer-thick sagittal sections of the medial compartment of the knee were used for staining. The slides were processed for H&E, safranin O, and fast green staining. TRAP staining was performed using a standard protocol (Sigma-Aldrich, St. Louis, MO, USA).

### Immunocytochemistry and histomorphometry

Knee joints were dissected from mice, the attached muscle was carefully removed and the tissues were fixed overnight with 10% formalin at 4 °C. After washing three times with ice-cold PBS, the samples were decalcified at 4 °C using 10% EDTA (pH 7.4) for 21 days and then embedded in paraffin. Four-micrometer-thick sagittal sections of the medial compartment of the knee were used for staining. The sections were stained with individual primary antibodies against COX-2 (ab15191, 1:100, Abcam), osterix (ab22552, 1:100, Abcam), OCN (M137, 1:100, Takara Bio Inc., Kusatsu, Shiga Prefecture, Japan), pSmad2/3 (sc-11769, 1:50, Santa Cruz Biotechnology Inc., Dallas, TX, USA), and MMP13 (ab3208, 1:50, Abcam) at 4 °C overnight. We used the horseradish peroxidase–streptavidin detection system (Dako) to detect the immunoreactivity. Then, we counterstained the sections with hematoxylin (Sigma-Aldrich). We counted the number of positively stained cells in four random visual fields of the subchondral bone in five sequential sections from each mouse in each group. For OCN staining, we normalized the number of positively stained cells to the number of cells per millimeter of adjacent bone surface (N·mm^−1^) in subchondral bone.

### Calcein double-labeling

To examine dynamic bone formation, we injected mice intraperitoneally with 0.08% calcein (Sigma-Aldrich, 20 mg·kg^–1^ b.w.) 8 and 2 days before euthanasia. We observed calcein double labeling in undecalcified bone slices under a fluorescence microscope. Four randomly selected visual fields of the distal metaphysis of the femur were measured to evaluate trabecular bone formation in subchondral bone.

### Von Frey testing

We detected mechanical sensitivity by applying 0.008-g von Frey filaments (Stoelting Co., Wood Dale, IL, USA) to the plantar surface of the hind paws of the mice. We used the number of paw withdrawals over three sets of 10 stimulations to represent the mechanical allodynia level.

### Statistics

The data are presented as the means ± standard deviations. For comparisons between two groups, two-tailed Student’s *t*-test was used. For comparisons among multiple groups (e.g., OARSI scores, bone mass, and microarchitecture among the groups), one-way ANOVA was used. All experiments were repeated at least three times, and representative experiments are shown. Differences were considered significant at *P* < 0.05. All data analyses were performed using SPSS, version 15.0, software (IBM Corp., Armonk, NY, USA).

## Supplementary information

Supplemental Figure

Supplemental Figure
