# Author Correction: Inhibition of cyclooxygenase-2 activity in subchondral bone modifies a subtype of osteoarthritis

**DOI:** 10.1038/s41413-025-00443-y

**Published:** 2025-12-08

**Authors:** Manli Tu, Mi Yang, Nanxi Yu, Gehua Zhen, Mei Wan, Wenlong Liu, Baochao Ji, Hairong Ma, Qiaoyue Guo, Peijian Tong, Li Cao, Xianghang Luo, Xu Cao

**Affiliations:** 1 Department of Orthopaedic Surgery, The Johns Hopkins University School of Medicine, Baltimore, MD 21205 USA; 2 Department of Endocrinology, The First Affiliated Hospital of Nanchang University, 330006 Nanchang, Jiangxi China; 3 Endocrinology Research Center of Xiangya Hospital, Central South University, 410008 Changsha, Hunan China; 4 Department of Orthopedic Surgery, The First Affiliated Hospital of Xinjiang Medical University, 830054 Urumqi, Xinjiang Uygur Autonomous Region China; 5 Department of Orthopedic Surgery, The First Affiliated Hospital of Zhejiang Chinese Medical University, 310006 Hangzhou, Zhejiang Province China

Correction to: *Bone Res* 10.1038/s41413-019-0071-x, published online 11 Sep 2019

Following publication of the original article,^1^ the authors regrettably found an error in Fig. 6a. The incorrect figure was included in the article inadvertently during figure preparation. Although these figures do not affect the results and conclusion in the article, all the authors agree to correct this negligence by providing corrected figure, to guarantee the accuracy of the article.

The originally published Fig. 6 was:

**Fig. 6:**
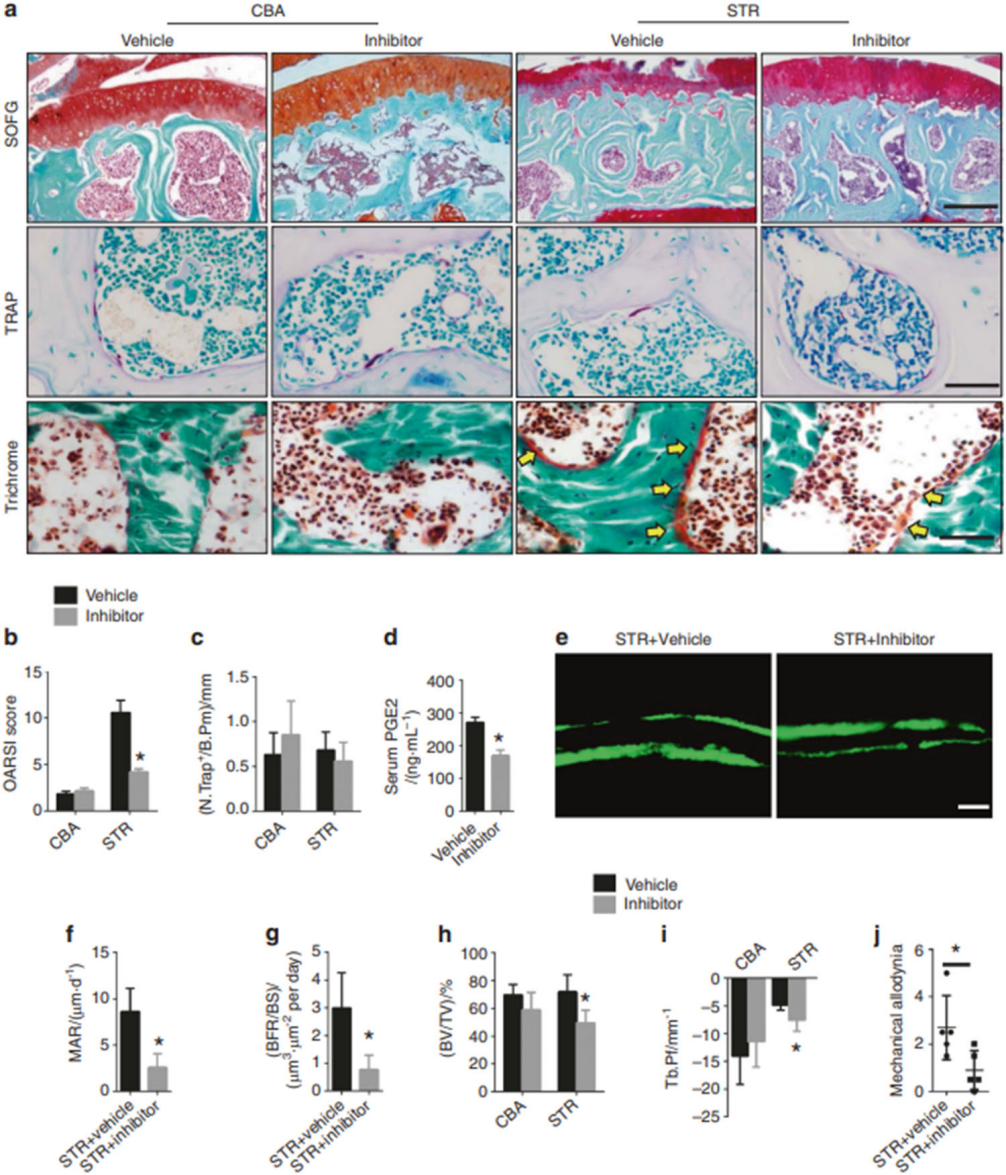
Inhibition of COX-2 attenuates spontaneous OA progression in STR/ORT mice. **a** Top: safranin O and fast green staining of the subchondral bone of STR/ORT mice and CBA control mice treated with vehicle or a COX-2 inhibitor. Proteoglycan (red) and bone (blue). Scale bar, 200 μm. Middle: representative images of TRAP staining in the subchondral bone of STR/ORT and CBA mice treated with vehicle or a COX-2 inhibitor. Scale bars, 50 μm. Bottom: trichrome staining of the subchondral bone of STR/ORT and CBA mice treated with vehicle or a COX-2 inhibitor. Scale bars, 50 μm. **b** OARSI scores of STR/ORT mice and CBA control mice treated with vehicle or a COX-2 inhibitor. *N* = 5 mice in each group from three independent experiments. **c** Quantitative analysis of TRAP^+^ cells on the bone surface in STR/ORT mice and CBA control mice treated with vehicle or a COX-2 inhibitor. *N* = 5 mice in each group from three independent experiments. **d** Serum PGE2 levels in STR/ORT mice and CBA control mice treated with vehicle or a COX-2 inhibitor. *N* = 5 mice in each group from three independent experiments. **e–g** Representative images of calcein double labeling of subchondral bone **e** and the quantification of the mineral apposition rate (MAR) **f** and bone formation rate (BFR) per bone surface (BS) **g** in STR/ORT mice treated with vehicle or a COX-2 inhibitor. Scale bar, 25 μm. **h** and **i** Quantitative analysis of the bone volume (BV)/tissue volume (TV) ratio **h** and trabecular pattern factor (Tb.Pf) **i** in subchondral bone, as determined by μCT analysis. *N* = 5 mice in each group from three independent experiments. j Quantitative analysis of mechanical allodynia in STR/ORT mice treated with vehicle or a COX-2 inhibitor, as measured by the foot-lift response frequency to stimulation with a 0.008-g von Frey filament. *N* = 5 mice in each group from three independent experiments. All data are shown as the mean ± standard deviation. *P < 0.05. Statistical significance was determined by Student’s *t*-test

The correct Fig. 6 should be:

**Fig. 6:**
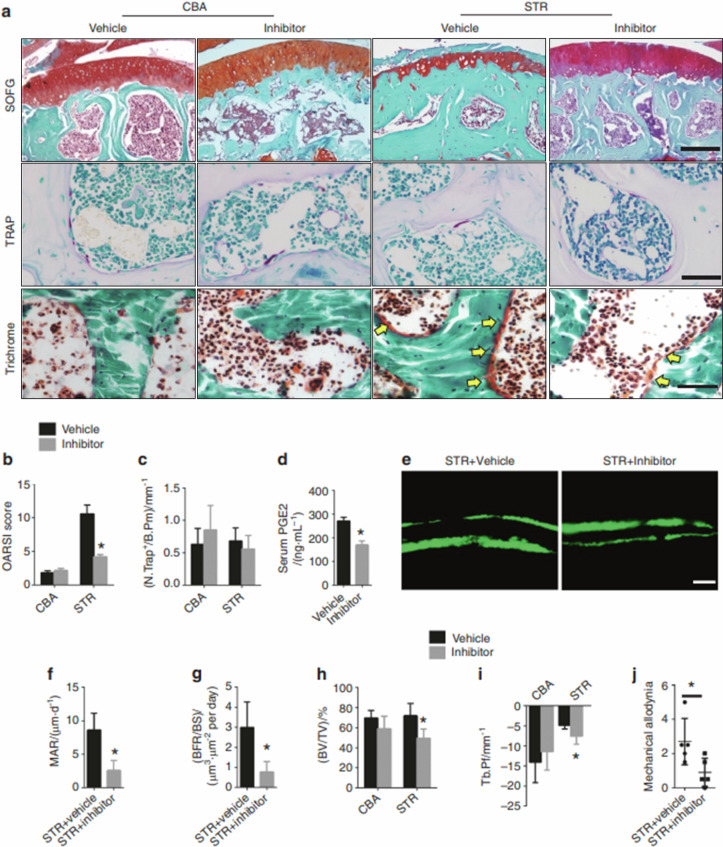
Inhibition of COX-2 attenuates spontaneous OA progression in STR/ORT mice. **a** Top: safranin O and fast green staining of the subchondral bone of STR/ORT mice and CBA control mice treated with vehicle or a COX-2 inhibitor. Proteoglycan (red) and bone (blue). Scale bar, 200 μm. Middle: representative images of TRAP staining in the subchondral bone of STR/ORT and CBA mice treated with vehicle or a COX-2 inhibitor. Scale bars, 50 μm. Bottom: trichrome staining of the subchondral bone of STR/ORT and CBA mice treated with vehicle or a COX-2 inhibitor. Scale bars, 50 μm. **b** OARSI scores of STR/ORT mice and CBA control mice treated with vehicle or a COX-2 inhibitor. *N* = 5 mice in each group from three independent experiments. **c** Quantitative analysis of TRAP^+^ cells on the bone surface in STR/ORT mice and CBA control mice treated with vehicle or a COX-2 inhibitor. *N* = 5 mice in each group from three independent experiments. **d** Serum PGE2 levels in STR/ORT mice and CBA control mice treated with vehicle or a COX-2 inhibitor. *N* = 5 mice in each group from three independent experiments. **e–g** Representative images of calcein double labeling of subchondral bone **e** and the quantification of the mineral apposition rate (MAR) **f** and bone formation rate (BFR) per bone surface (BS) **g** in STR/ORT mice treated with vehicle or a COX-2 inhibitor. Scale bar, 25 μm. **h** and **i** Quantitative analysis of the bone volume (BV)/tissue volume (TV) ratio **h** and trabecular pattern factor (Tb.Pf) **i** in subchondral bone, as determined by μCT analysis. *N* = 5 mice in each group from three independent experiments. j Quantitative analysis of mechanical allodynia in STR/ORT mice treated with vehicle or a COX-2 inhibitor, as measured by the foot-lift response frequency to stimulation with a 0.008-g von Frey filament. *N* = 5 mice in each group from three independent experiments. All data are shown as the mean ± standard deviation. *P < 0.05. Statistical significance was determined by Student’s *t*-test

The original article^1^ has been updated.

## References

[1] Tu, M. et al. Inhibition of cyclooxygenase-2 activity in subchondral bone modifies a subtype of osteoarthritis. *Bone Res.* **7**, 29, 10.1038/s41413-019-0071-x (2019). 31666999 10.1038/s41413-019-0071-x PMC6804921

