# Supplementary material for: Inhibition of cyclooxygenase-2 activity in subchondral bone modifies a subtype of osteoarthritis
Source: Bone Res. 2019 Sep 11;7:29. doi: 10.1038/s41413-019-0071-x (PMC6804921; doi:10.1038/s41413-019-0071-x)

**Supplementary Materials:**

**Supplemental Figure 1.** Elevated COX-2 expression in cortical bone and trabecular bone of STR/ort mice. **(a-b)** Immunohistochemical staining **(a)** and quantitative analysis **(b)** of COX-2^+^ cells (brown) in tibia cortical bone of 6 months old STR/ort and CBA mice. Scale bars, 50 μm. N = 5 per group. **(c-d)** Immunohistochemical staining **(c)** and quantitative analysis **(d)** of COX-2^+^ cells (brown) in tibia trabecular bone of 6 months old STR/ort and CBA mice. Scale bars, 50 μm. N = 5 per group. All data shown as mean ± standard deviation. *P < 0.05; **P < 0.01. Statistical significance was determined by Student t-test.

**
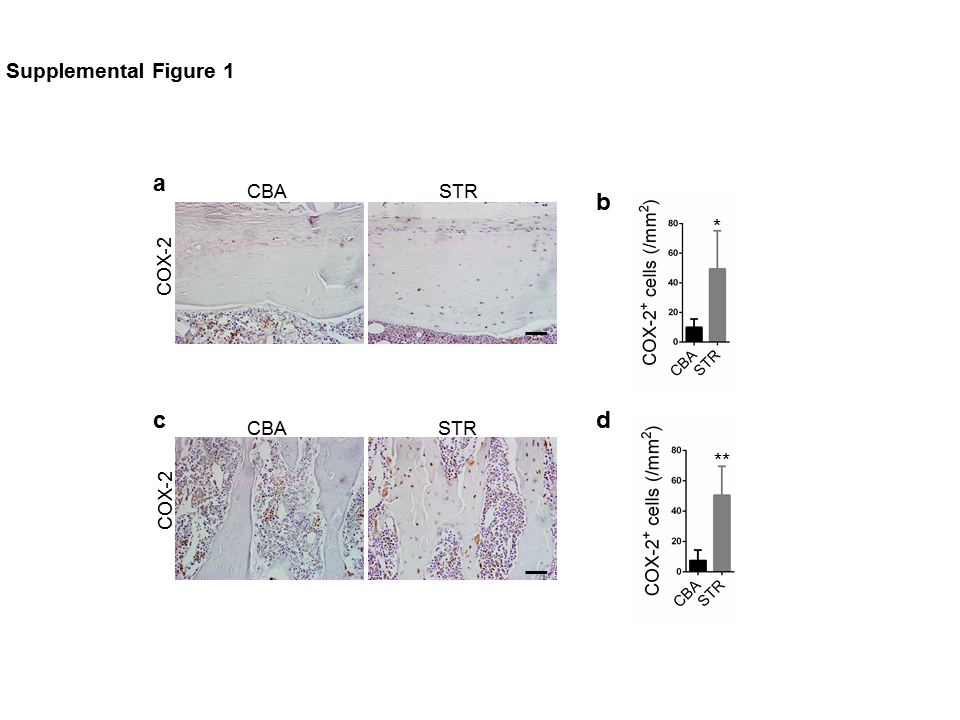
**

**Supplemental Figure 2.** Uncoupled subchondral bone remodeling was increased in TNF-α transgenic RA mice. **(a)** Representative images of TRAP staining, immunohistochemical staining for osteocalcin (OCN) and osterix (OSX) in subchondral bone marrow of TNF-α Tg^+/-^ mice and their wild type (WT) controls at 4 months old. Scale bars, 25 μm. **(b)** Quantitative analysis of TRAP^+^ cells in bone surface of TNF-α Tg^+/-^ mice and their WT controls at 4 months old. **(c)** Quantitative analysis of OCN^+^ cells in bone surface of TNF-α Tg^+/-^ mice and their WT controls at 4 months old. **(d)** Quantitative analysis of OSX^+^ cells (per mm^2^) in subchondral bone marrow of TNF-α Tg^+/-^ mice and their WT controls at 4 months old. All data shown as mean ± standard deviation. *P < 0.05. Statistical significance was determined by Student t-test.


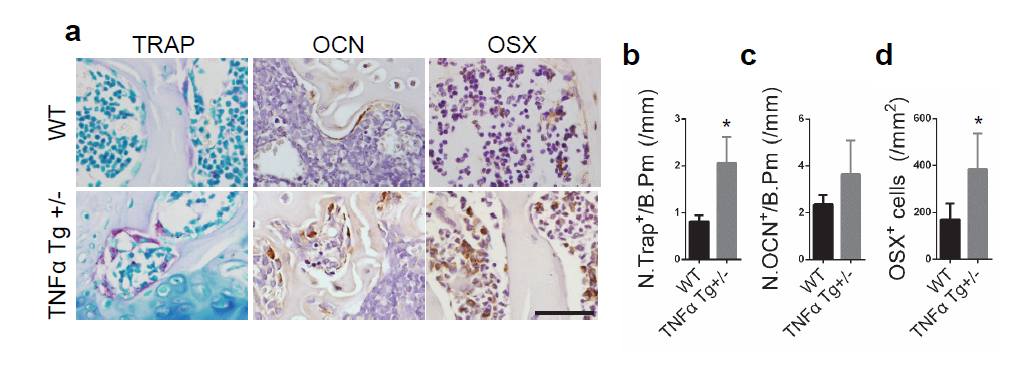


**Supplemental Figure 3.** Conditional knockout of COX-2 in osteocytes of TNF-α transgenic RA mice rescues uncoupled subchondral remodeling. **(a)** Representative images of TRAP staining, immunohistochemical staining for osteocalcin (OCN) and osterix (OSX) in subchondral bone of COX-2^flox/flox^, TNF-α Tg^+/-^ -COX-2^flox/flox^, and TNF-α Tg^+/-^ DMP1-Cre:: COX-2^flox/flox^ mice. Scale bars, 25 μm. **(b-d)** Quantitative analysis of TRAP^+^ cells **(b)**, OCN^+^ cells **(c)**, and OSX^+^ cells **(d)** in subchondral bone of COX-2^flox/flox^, TNF-α Tg^+/-^ -COX-2^flox/flox^, and TNF-α Tg^+/-^ DMP1-Cre:: COX-2^flox/flox^ mice. N = 5 per group. All data shown as mean ± standard deviation. *P < 0.05; **P < 0.01. Statistical significance was determined by ANOVA.


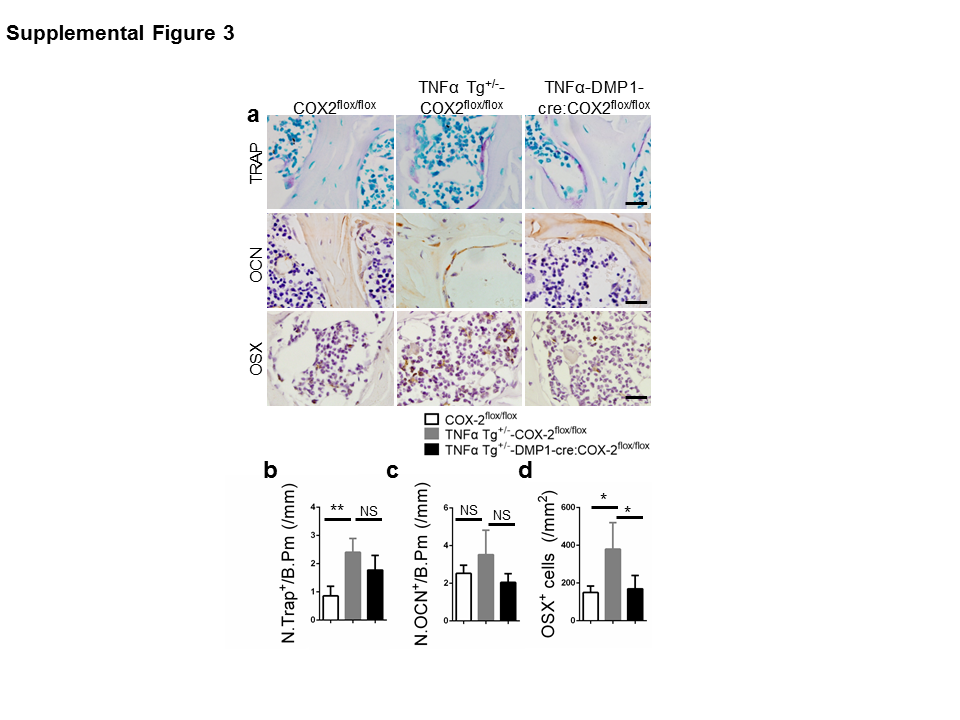


**Supplemental Figure 4.** Inhibition of COX-2 in TNF-α transgenic RA mice rescues uncoupled subchondral remodeling. **(a)** Safranin O and fast green staining of sagittal sections of the tibia medial subchondral bone of Tg^+/-^ mice treated with vehicle (Vehicle) and TNF-α Tg^+/-^ mice treated with COX-2 inhibitor (Inhibitor). Scale bars, 200 μm. **(b)** Serum PGE2 levels of TNF-α transgenic RA mice treated with vehicle or COX-2 inhibitor. N = 5 per group. **(c-d)** Quantitative analysis of bone volume (BV) per tissue volume (TV) **(c)** and trabecular pattern factor (Tb.Pf) **(d)** in subchondral bone of Vehicle and Inhibitor groups. N = 5 per group. **(e)** Representative images of TRAP staining and immunohistochemical staining for OCN and OSX in subchondral bone of Vehicle, Inhibitor and wild type (WT) controls. Scale bars, 25 μm. **(f-h)** Quantitative analysis of TRAP^+^ cells **(f)**, OCN^+^ cells **(g)**, and OSX^+^ cells **(h**) in subchondral bone of Vehicle, Inhibitor and wild type (WT) controls. N = 5 per group. All data shown as mean ± standard deviation. *P < 0.05; **P < 0.01. Statistical significance was determined by Student t-test for **(b-d)**. Statistical significance was determined by ANOVA for **(f-h)**.


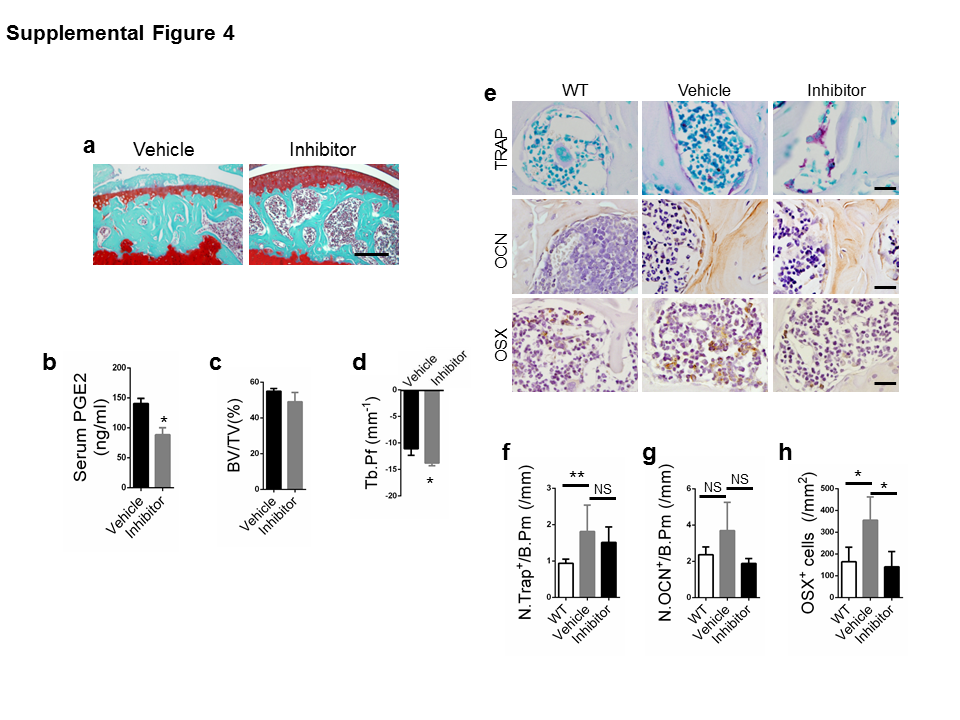


**Supplemental Figure 5.** STR/Ort mice have a high bone mass phenotype.

**(a-f)** Representative microcomputed tomography (μCT) images **(a)** and quantitative μCT analysis **(b-f)** of trabecular bone and cortical microarchitecture in femora from 6 months old STR/Ort and CBA mice. (Tb. BV/TV, trabecular bone volume per tissue volume; Tb.N, trabecular number; Tb.Th, trabecular thickness; Tb.Sp, trabecular separation; Ct.Th, cortical thickness.). N = 5 per group. All data shown as mean ± standard deviation. *P < 0.05; **P < 0.01. Statistical significance was determined by Student t-test.


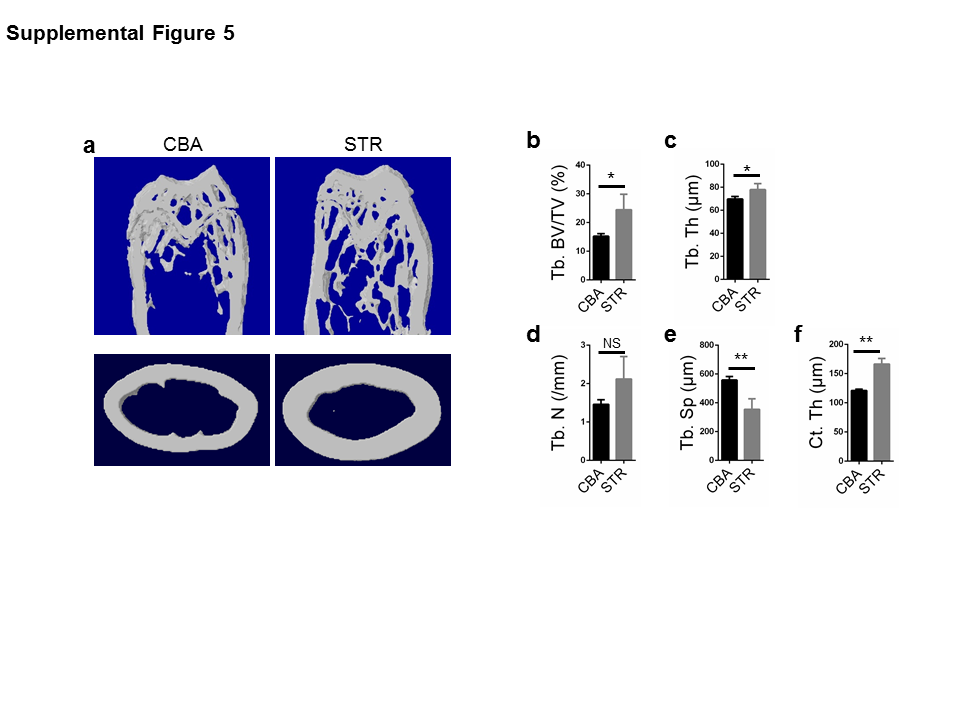

Supplement: Supplementary file 1 — Supplemental Figure [file 41413_2019_71_MOESM1_ESM.docx]
