# Supplementary material for: Inhibition of cyclooxygenase-2 activity in subchondral bone modifies a subtype of osteoarthritis
Source: Bone Res. 2019 Sep 11;7:29. doi: 10.1038/s41413-019-0071-x (PMC6804921; doi:10.1038/s41413-019-0071-x)

Supplemental Figure 1

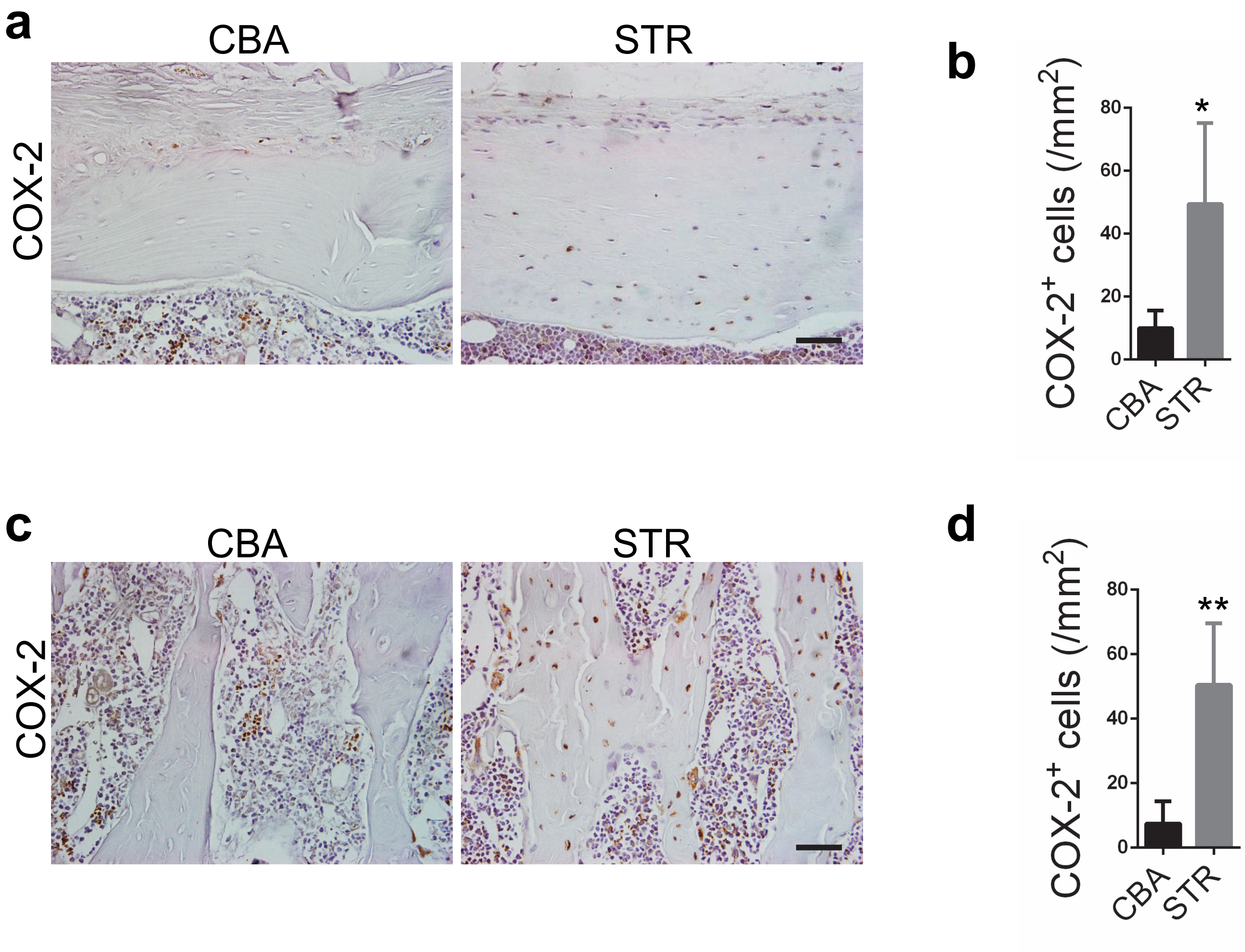

Supplemental Figure 2

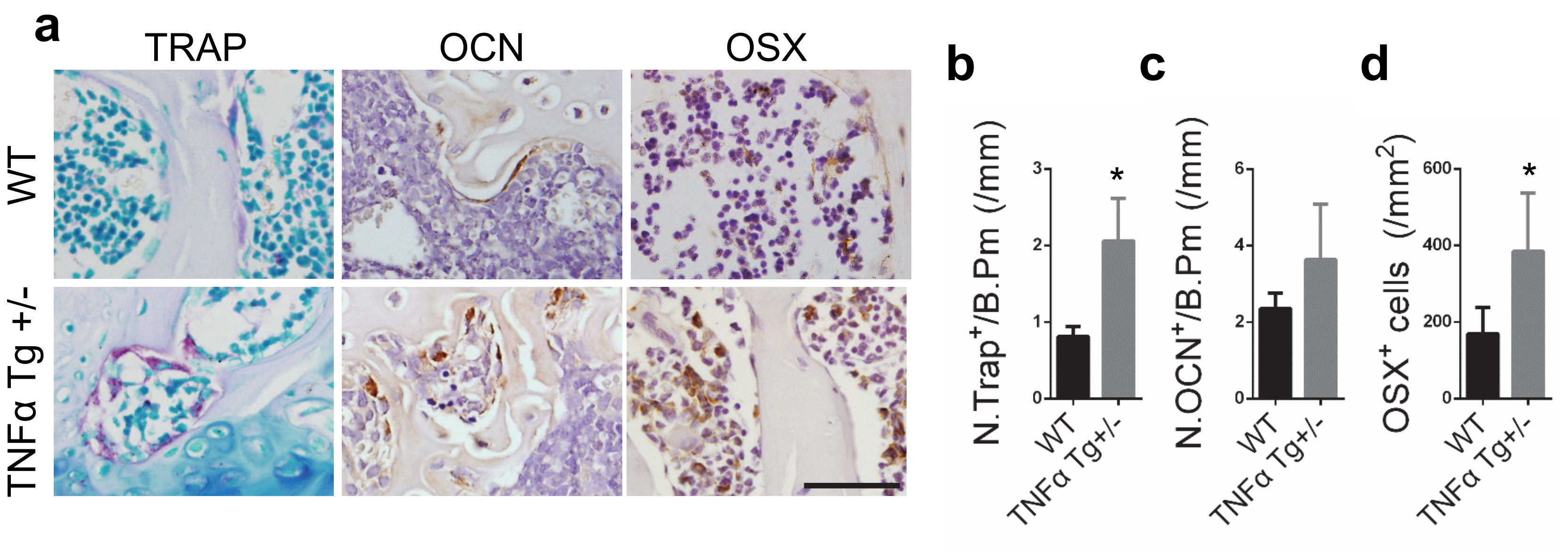

Supplemental Figure 3

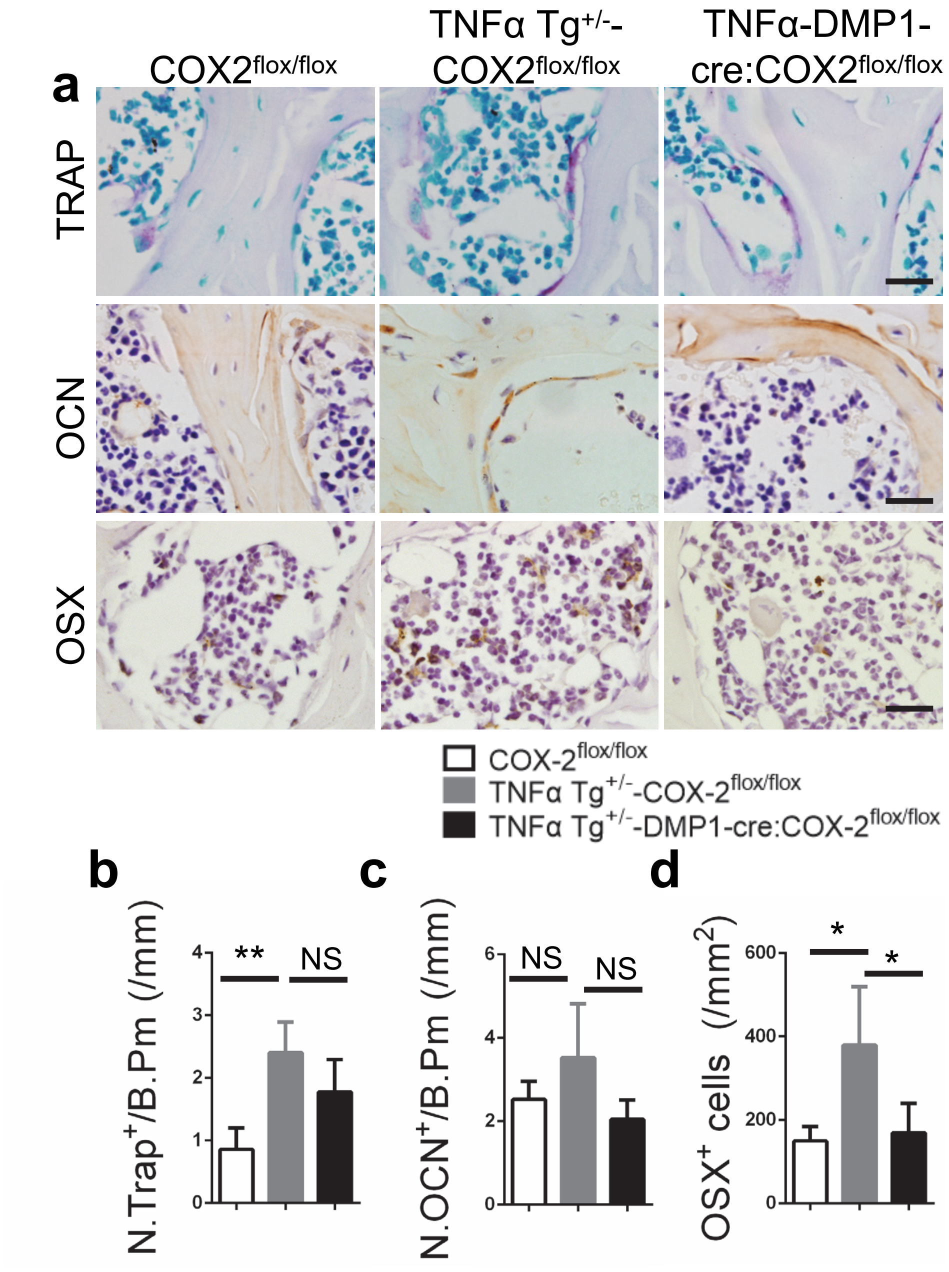

Supplemental Figure 4

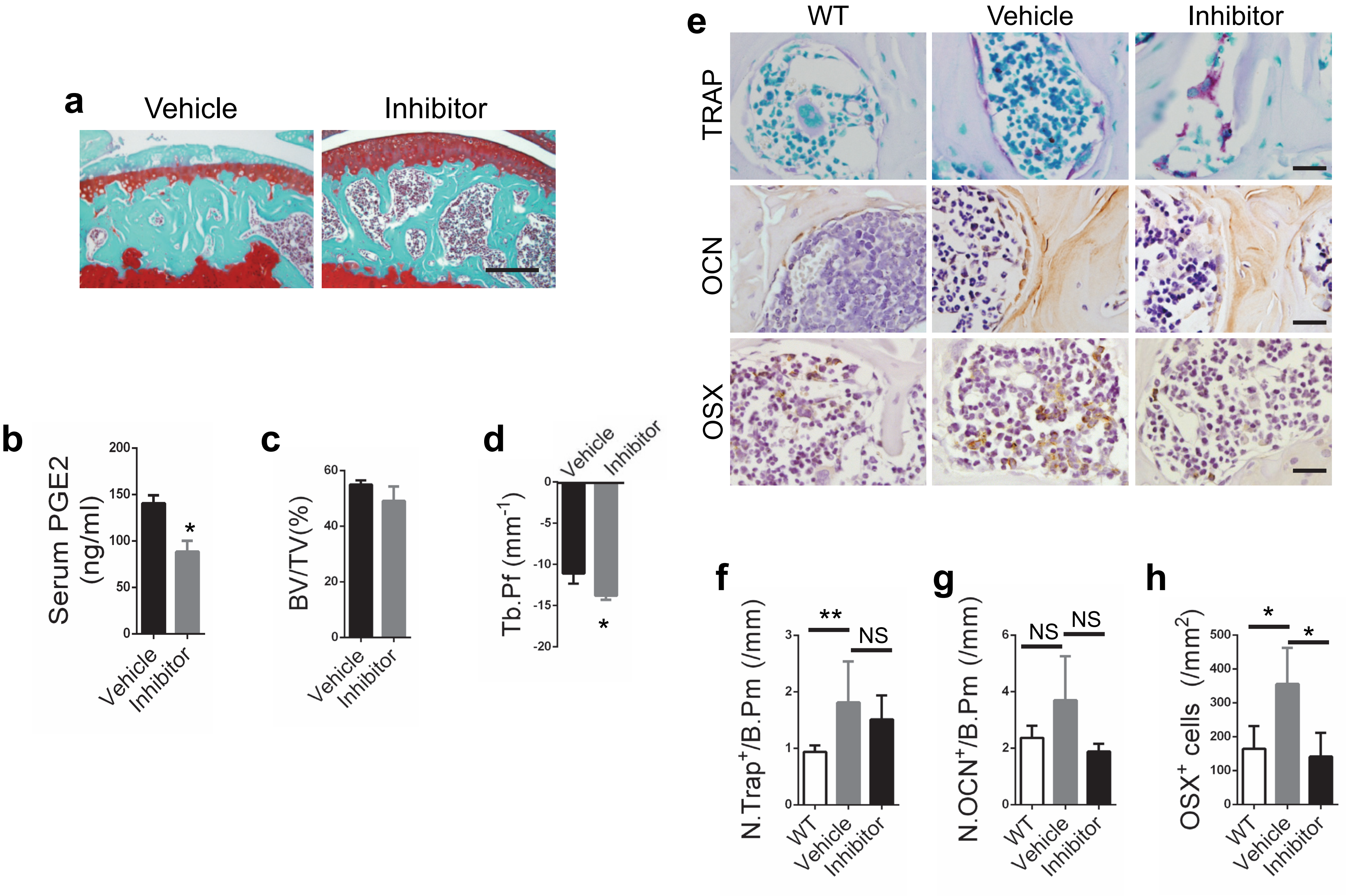

Supplemental Figure 5

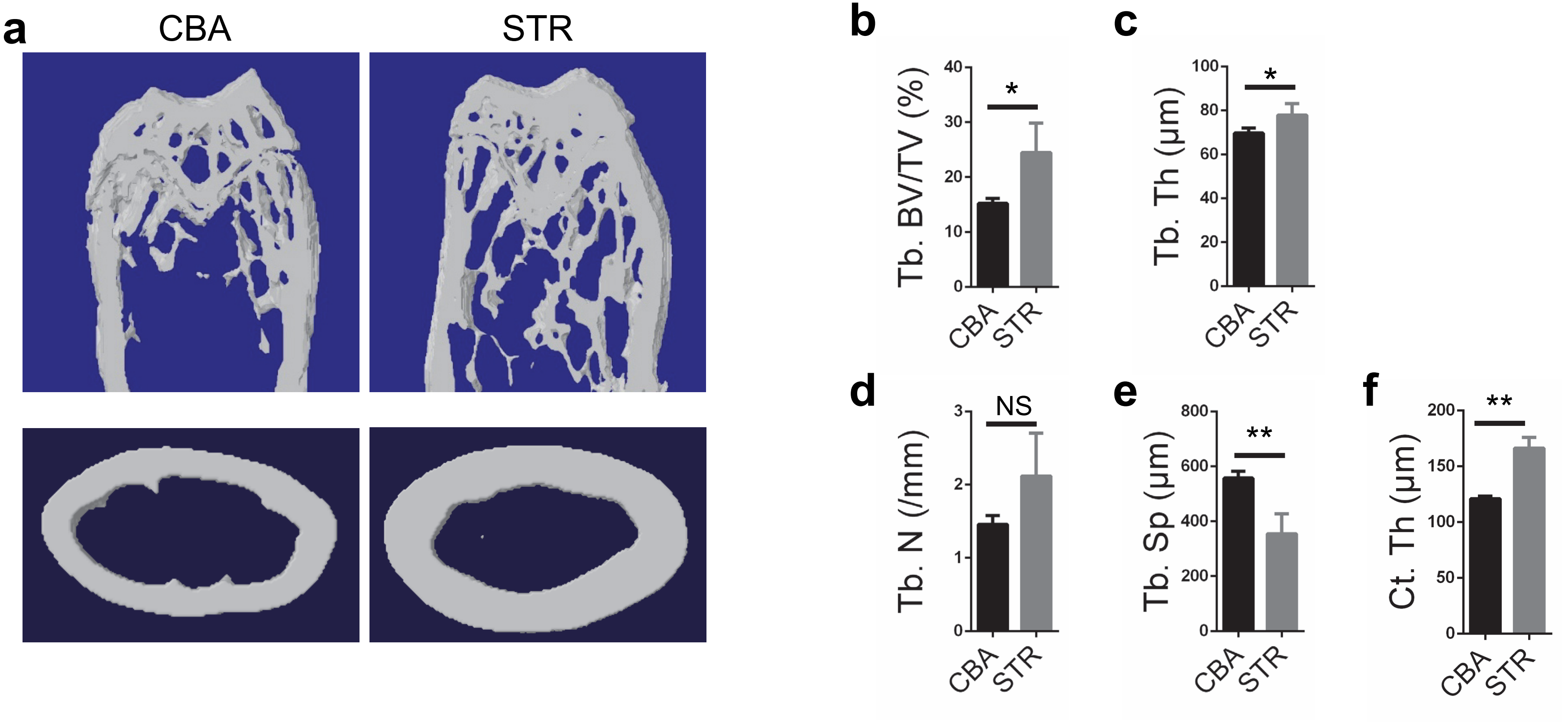

Supplement: Supplementary file 2 — Supplemental Figure [file 41413_2019_71_MOESM2_ESM.pdf]
